# Genomic structural variation in tomato and its role in plant immunity

**DOI:** 10.1186/s43897-022-00029-w

**Published:** 2022-03-10

**Authors:** Emma Jobson, Robyn Roberts

**Affiliations:** 1 Montana State University Extension, Montana State University, Bozeman, MT 59717 United States; 2 Agricultural Biology Department, College of Agricultural Sciences, Colorado State University, Fort Collins, CO USA

**Keywords:** Immunity, Structural variation, Tomato, Genetic engineering, Disease, Sequencing

## Abstract

It is well known that large genomic variations can greatly impact the phenotype of an organism. Structural Variants (SVs) encompass any genomic variation larger than 30 base pairs, and include changes caused by deletions, inversions, duplications, transversions, and other genome modifications. Due to their size and complex nature, until recently, it has been difficult to truly capture these variations. Recent advances in sequencing technology and computational analyses now permit more extensive studies of SVs in plant genomes. In tomato, advances in sequencing technology have allowed researchers to sequence hundreds of genomes from tomatoes, and tomato relatives. These studies have identified SVs related to fruit size and flavor, as well as plant disease response, resistance/susceptibility, and the ability of plants to detect pathogens (immunity). In this review, we discuss the implications for genomic structural variation in plants with a focus on its role in tomato immunity. We also discuss how advances in sequencing technology have led to new discoveries of SVs in more complex genomes, the current evidence for the role of SVs in biotic and abiotic stress responses, and the outlook for genetic modification of SVs to advance plant breeding objectives.

## Introduction

Studies exploring genetic variation have built foundational knowledge in genetics, molecular biology, cell biology, breeding, and evolution in plants, animals, and microorganisms. The methods applied, and the ability to capture the true genetic variation present in species, has evolved as technology has advanced. The power to sequence whole genomes with increased efficiency and decreased cost has allowed researchers to begin to understand new levels of genomic variation. Recently, the ability to accurately sequence long regions of genomes and single molecules has illustrated the impact of larger variations across and within genomes, referred to as structural variants (SVs).

**Fig. 1:**
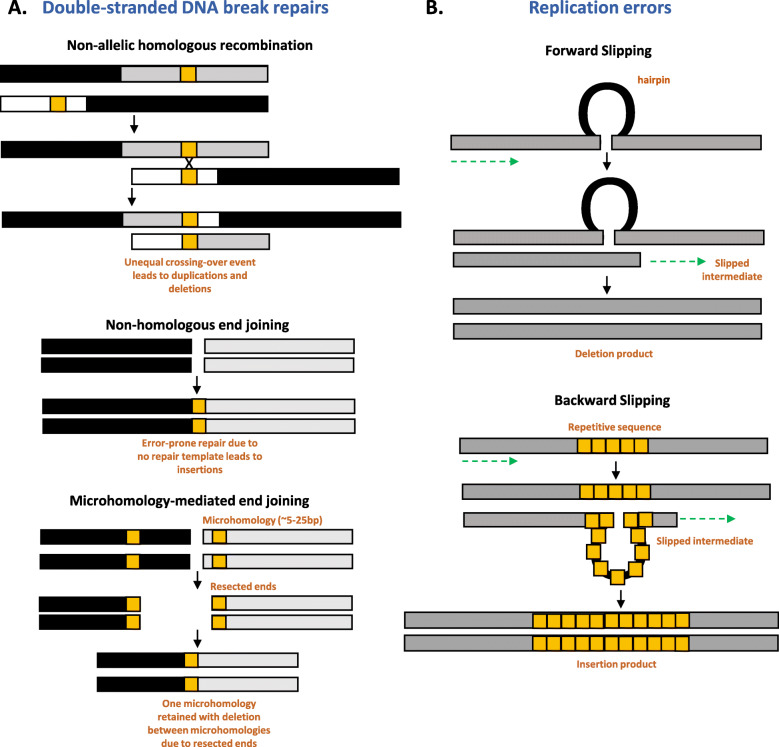
Structural variants are derived from a variety of molecular mechanisms. Examples of **A**) double-stranded DNA break repair and **B**) replication error mechanisms that may lead to structural variation within genomes. Black and gray regions represent chromosomal regions. Gold indicates areas of homology (**A**) or repetitive sequence (**B**)

Any deviation from the reference genome for a particular species can be described as ‘structural variation,’ but generally structural variants are defined as changes larger than a few bases, which can range from 30 base pairs to several megabases. Insertions, deletions, translocations, inversions, changes in copy numbers, tandem duplications, and presence/absence deviations are all examples of structural variation (Alkan et al., 2011; Sudmant et al., 2015; Sedlazeck et al., 2018). Structural variants (SVs) are derived from equally diverse genomic mechanisms, including non-allelic homologous recombination, non-homologous end joining, microhomology mediated end joining, errors in replication, and serial replication slippage (Fig. 1). Mobile genetic elements that can move within a genome, such as transposons, endogenous viral elements, and satellite DNAs, can also cause structural genomic changes resulting in SVs (Keidar-Friedman et al., 2020; Kirov et al., 2020; Vicient & Casacuberta, 2020).

The significance and prevalence of SVs was first identified in human studies in the early 2000s, soon after the human genome was sequenced. Genomic repetitions were found to play a role in the development of Parkinson’s as well as Huntington’s diseases (Stankiewicz & Lupski, 2010; Schule et al., 2017; McColgan & Tabrizi, 2018). Various SVs have been linked to cancer, including amplification of genes which inactivate BRCA1 and BRCA2 (Friedman et al., 1994; Wooster et al., 1995). Multiple studies in humans have indicated that SVs contribute to more genetic variation than single nucleotide variations (SNVs). Huddleston et al. determined that SVs account for 3.4 times more genomic variation than SNVs (Huddleston et al., 2017). Pang et al., estimated that the average genomic variation between two humans due to SNVs is 0.1% compared 1.5% due to SVs (Pang et al., 2010). These studies serve as examples of the vast potential of SVs as agents of genomic change. However, while SVs have been linked to many chronic illnesses of humans, the role of SVs in plants is only beginning to be revealed.

Compared to mammalian genomes, plant genomes typically contain many more repetitive sequences that originate from spontaneous genome duplications (autopolyploidy), cross-species chromosomal hybridizations (allopolyploidy), and ancient duplication events (paleopolyploidy). Additionally, selective breeding and crop improvement have led to the development of polyploid genomes, such as hexaploid bread wheat (*Triticum aestivum*), which is comprised of > 80% repetitive sequence and is derived from three separate genomes to contain 3 copies of each chromosome. Plants also contain transposable elements which can play a large role in SVs, as is likely the case in maize. Although SVs can occur throughout the genome, studies suggest that their distribution is nonrandom, and there is a higher prevalence of SVs in centromeric and subtelomeric regions (Turner et al., 2008).

An analysis of 3000 rice genomes identified over 63 million structural variants and classified each SV as either an insertion, deletion, inversion, or duplication (Fuentes et al., 2019). Based on these designations researchers determined that deletions were the most prevalent form of SVs, followed by insertions and duplications; inversions were the least common SV identified in this study. Furthermore, this study found that long SVs were enriched in promoter regions, and shorter SVs were clustered in the 5′ UTR (Fuentes et al., 2019).

In tomato, the development of a reference genome and subsequent comparison and analysis of thousands of tomato genomes has led to improved understanding of molecular mechanisms and biological models. For example, two studies that analyzed over 1000 tomato genomes (Blanca et al., 2015) and 360 tomato genomes (Lin et al., 2014) gave insights into tomato domestication, demonstrating that domestication occurred in two steps. The small-fruited wild progenitor of tomato, *Solanum pimpinellifolium,* was originally domesticated to cherry tomato (*Solanum lycopersicum var. cerasiforme*) in the Andes. Cherry tomato moved across the region, and in Mesoamerica cherry tomato was improved and selected for larger fruit size and mass that led to the large-fruited tomato (*Solanum lycopersium*). Recent advances in sequencing capabilities have also made way for the development of a tomato pan-genome (Gao et al., 2019), which revealed the genetic diversity among 725 accessions and nearly 5000 new genes absent from the ‘Heinz 1706’ reference genome, which were highly enriched in defense response genes.

Considering the recent advances in our understanding of tomato natural variation, genomic variation, and improved sequencing technology, this review focuses on the known roles of structural variants in plant immunity, particularly in tomato, and the current gaps in knowledge. We also discuss how SVs influence important agronomic traits, such as disease response, and the prospects of utilizing genetic engineering to introduce structural variants as a tool for germplasm improvement.

### Advances in genome sequencing have made it possible to identify SVs

Innovations in sequencing technology and computational biology have greatly impacted our ability to detect and understand the full diversity of genetic variation. Initial studies to characterize genomic differences were performed at the chromosomal level using microscopes (Feuk et al., 2006; Yuan et al., 2021). In 1953, Watson and Crick, with Rosalind Franklin’s x-ray crystallography image, described the three-dimensional structure of double stranded DNA which provided the basis for genomic work moving forward (Watson & Crick, 1953). It wasn’t until 1977 that researchers were able to “read” a genomic sequence; Sanger sequencing, named for Fred Sanger who developed the technique, relied on chain termination to sequentially arrange the nucleotides (Sanger et al., 1977; Heather & Chain, 2016). In 1990, the Human Genome project began, and in 1996 *Saccharomyces cerevisiae* was the first fully sequenced eukaryotic organism. The first plant, *Arabidopsis thaliana,* was sequenced in 2000. More than 1000 plant species have been fully sequenced as part of the 1KP project (1000 Plants Project), and the number of species sequenced continues to grow exponentially (Chen et al., 2018; Soltis & Soltis, 2021). However, current assembled genomes exist for only about 1% of all plant species (Soltis & Soltis, 2021), demonstrating how much we still have to learn about plant genomes, molecular mechanisms of the genetics, and the roles and impacts of structural variation on plants. New initiatives are rapidly expanding genome sequencing in plants. The 10,000 Plant Project, started in 2017 at the International Botanical Congress, aims to sequence 10,000 plant species over the next several years, and the Darwin Tree of Life project aims to sequence all 70,000 eukaryotic organisms in Britain and Ireland.

While advancing genome sequencing techniques, researchers also became aware of the vast amount of variation within genomes, and how to best capture and characterize those variants. As the Human Genome Project was nearing completion, the project shed new insight on another common form of genetic variation, single nucleotide polymorphisms (SNPs), at the genome scale (Vignal et al., 2002; Weiner & Hudson, 2002). For the next two decades, SNP-based molecular markers played a monumental role building the foundation to understand underlying genetic mechanisms associated with traits of interest.

Improvements in technology have dramatically increased the usage of sequencing-based computational analysis. The introduction of both short- and long- read sequencing technology and the development of efficient genome assembly pipelines has allowed researchers to collect vast amounts of genomic data. Identifying SVs using sequencing-based computational analysis relies on four different strategies which may be used individually or in combination. Short reads sequencing is employed for paired-end mapping, split-read mapping, read depth, and de novo assembly. This data is then complemented with long read sequencing data which allows researchers to identify large chromosomal arrangements. The combination of both short and long read sequencing dramatically improves the coverage and depth of genomic assembly.

The introduction of short read sequencing allowed researchers to begin identifying single nucleotide polymorphisms (SNPs) (Vignal et al., 2002; Weiner & Hudson, 2002). Correlating SNPs with specific traits allowed researchers to develop molecular markers to track specific traits within a population. While SNPs were primarily identified using short read sequencing (SRS, 75–400 bp) from Next Generation Sequencing technology (such as Illumina’s HiSeq platform) or Sanger sequencing, short read sequences do not permit the assembly of repetitive sequences due to the lack of sufficient overlap of DNA sequences. Additionally, SRS preferentially amplifies repetitive sequences, so it is difficult or impossible to piece apart artifacts from real repetitive sequences.

The early strategies for detecting SVs were SNP arrays and array comparative genomic hybridization (array-CGH). Array-CGH relies on comparative hybridization of the reference and test samples to hybridization targets. The signal ratio is then used as a proxy for estimating copy number variation. SNP arrays also utilize hybridization to detect genomic variation. However, SNP probes are designed to capture unique differences between samples. This increases the sensitivity for SNP arrays to detect copy number variations and unique alleles. However, SNP arrays do have increased background noise compared to array-CGH (Alkan et al., 2011). Both strategies are well suited for high throughput analysis, but are unable to detect all forms of SVs, especially smaller variations and breakpoints. Arrays are also limited to only detecting differences in the sequences represented by the probes, and are primarily designed for use in diploid organisms (Alkan et al., 2011).

Recently, advancements in long read sequencing technology (LRS, Third Generation Sequencing, 5–30 kb) now allow researchers to identify larger genomic variants up to several megabases in length. LRS amplifies a single DNA molecule, which avoids amplification bias of repetitive sequences and generates enough DNA overlaps to assemble whole chromosomes. Single-molecule real-time (SMRT) sequencing, such as Pacific Bioscience’s Sequel platform, and nanopore sequencing, such as Oxford Nanopore’s MinION platform, are available long-read sequencing methods. While SRS typically has lower error rates and therefore more accurately calls SNPs compared to LRS, LRS is essential for de novo genome assembly and chromosome-level sequencing.

The plunging costs of sequencing have made identifying SVs less cost-prohibitive, and the development of ‘in-house’ long-read sequencers such as Oxford Nanopore’s MinION platform brings costs down significantly. The development of easy-to-use genomic DNA preparation, library, and barcoding kits have made sequencing broadly accessible to biologists.

Published in 2009, maize became the first plant genotyped for SV analysis (Springer et al., 2009). Maize is a phenotypically diverse and genetically complex crop. It’s estimated that there is a higher frequency of SNPs between two inbred lines of maize than there is between chimpanzees and humans (Buckler et al., 2006). Furthermore, structural variation at the chromosomal level in maize had also previously been demonstrated as part of the work performed by Barbara McClintock and others (Brown, 1949; McClintock et al., 1981; Adawy et al., 2004). This previous knowledge made maize an excellent candidate for SV analysis. Using array-CGH, Springer et al were able to detect several hundred copy number variations and several thousand examples of presence/absence variations between two inbred maize lines, once again illustrating the extreme prevalence and potential impact of SVs (Springer et al., 2009).

Hundreds of other studies have since investigated the impact of SVs on plant growth and development. As more non-model species, wild relatives, and additional varieties/accessions are sequenced, and pangenomes and additional reference-level genomes are developed, it is likely that SV discoveries that impact plant growth and development will continue to uncover additional impactful variants. A recent study estimated that one-third of domestication alleles are the result of an SV (Gaut et al., 2018), suggesting that many phenotypic changes in crops are associated with SVs.

## Evidence for the role of SVs in plant stress responses

### SVs in plants play a key role in plant growth and development, and abiotic stress

Numerous studies have shown that SVs can play an important role in plant growth and development. Examples include an indel in rice that influences root architecture (Uga et al., 2013), a tandem duplication in the *Reduced Height* gene in wheat plays a key role in plant height (Li et al., 2012), and a copy number variation in *Brassica napus* impacts flowering time (Schiessl et al., 2017). These examples represent only a small fraction of the diversity and prevalence of SVs in plants, and their role in growth and development.

Other studies have shown that SVs can play a critical role in a plant’s response to abiotic stresses, including temperature and elemental toxicity. Table 1 illustrates some of the more recent work focused on the role of SVs in plant stress response. In Arabidopsis, one study illustrated that copy number variations were found to play a key role in temperature response. Furthermore, this study found the CNV were enriched in transposable elements and stress genes, indicating that SVs may play an important role in a plant’s ability to adapt to different environments (DeBolt, 2010). In wheat, copy number variation of VRN-A1 impacts the frost tolerance of the plant (Zhu et al., 2014).

**Table 1:** Examples of plant genomic structural variants known to impact stress responses

Species	Biotic/Abiotic Stress	Type of SV	References
**Arabidopsis (*Arabidopsis thaliana*)**	Stress Response	Insertion/Deletion	(Lu et al., 2012)
	Temperature response and stress genes	Copy Number Variation	(DeBolt, 2010)
**Barley (*Hordeum vulgare*)**	Aluminum tolerance	Insertion	(Fujii et al., 2012)
	Boron toxicity	Copy Number Variation	(Sutton et al., 2007)
	Disease resistance	Copy Number Variation	(Munoz-Amatriain et al., 2013)
**Maize (*Zea mays*)**	Aluminum tolerance	Copy Number Variation	(Maron et al., 2013)
	Disease Resistance	Copy Number Variation	(Belo et al., 2010)
**Oilseed (*Brassica napus*)**	*Verticillium longisporum* resistance	Presence/Absence Variation	(Gabur et al., 2020)
**Rice (*Oryza sativa*)**	Disease Resistance	Copy Number Variation	(Xu et al., 2015)
**Scott’s Pine (*Pinus sylvestris*)**	Root rot resistance	Copy Number Variation	(Skipars et al., 2012)
**Sorghum (*Sorghum bicolor*)**	Disease resistance	Presence/Absence Variation; Copy Number Variation	(Zheng et al., 2011; Mace et al., 2014)
**Soybean (*Glycine max*)**	Nematode resistance	Copy Number Variation	(Cook et al., 2012; Bayless et al., 2016; Bayless et al., 2018; Bayless et al., 2019)
	Stress Response	Presence/Absence Variation; Copy Number Variation	(Huan et al., 2005; McHale et al., 2012)
	Disease Resistance	Copy Number Variation	(Lee et al., 2015)
**Tomato (*Solanum lycopersicum)***	Unknown	Various	(Gao et al., 2019)
**Wheat (*Triticum aestivum*)**	Frost tolerance	Copy Number Variation	(Zhu et al., 2014)
**Palmer amaranth (*Amaranthus palmeri*)**	Glyphosate resistance	Copy Number Variation	(Gaines et al., 2010)

SVs have also been shown to play a role in toxicity tolerance in many different species. In barley, aluminum tolerance is conferred by a 1 kb insertion (Fujii et al., 2012). Also in barley, a copy number variation of the BOR1 gene can create boron toxicity tolerance (Sutton et al., 2007). In maize, increased copy number of MATE1 results in superior aluminum tolerance (Maron et al., 2013).

Studies to identify potentially useful SVs in tomato have focused on a single gene or trait. Soyk et al. identified a tandem duplication in *sb1* and *sb3* that impacted branching patterns in tomato (Soyk et al., 2019a; Soyk et al., 2019b). Xu et al., discovered that a large inversion in *fas* was associated with larger fruit size (Xu et al., 2015). Similarly, Mu et al., determined that a deletion in the *CSR* gene also increased fruit weight (Mu et al., 2017). Muller et al., demonstrated that a deletion in *LNK2* can impact the circadian rhythm of tomatoes (Muller et al., 2018).

Beyond screening entire genomes for SVs, emerging technologies in genome editing have allowed researchers to assign causality to SVs, by recreating them in different accessions. For example, Alonge et al. used long read sequencing to capture 238,490 SVs across 100 diverse tomato genomes (Alonge et al., 2020); from this data they were able to identify SVs associated with flavor, fruit size, and productivity. They were then able to recreate specific phenotypes associated with an SV using the CRISPR/Cas9 gene editing system. Although there is limited research related to the impact of SVs on tomato growth and development, it has become a unique species for identifying SVs, and then recreating them using CRISPR-Cas9 genome editing tools.

### SVs are enriched in regions of the genome associated with disease response

In comparison to studies focused on growth and development, the impact of SVs on plant disease response is much less well understood. However, there have been a number of studies that have found that SVs are localized to regions of the genome associated with plant stress and defense.

In 2012 Lu et al. sequenced two varieties of Arabidopsis to investigate genomic variation. In total they captured 349,171 SNPS, 58,085 small indels, and 2315 large indels. After analysis, they determined that variations were enriched in regions of the genome associated with disease response (Lu et al., 2012). Similarly, in 2009 Belo et al. used array-CGH to analyze 13 lines of inbred maize and found that many CNVs were within or adjacent to a region responsible for disease response (Belo et al., 2010). In sorghum, Zheng et al., resequenced three varieties to discover nearly two million SNPs, indels, presence/absence variations, and CNVs. From this data, they determined that the majority of large effect variations were found in genes with leucine rich repeats, or disease resistance *R* genes (Zheng et al., 2011).

A study published by McHale et al. analyzed SVs in four soybean varieties (McHale et al., 2012). The relative abundance of SVs between the varieties was low, with the exception of regions of the genome associated with nucleotide binding and receptor like protein classes. These results suggested that SVs were colocalizing with regions of the genome associated with plant disease response.

Similar discoveries have been made in many other plant species. In *Brassica napus*, Dolatabadian et al., analyzed the resistance gene distribution in 50 lines. They characterized 1749 resistance genes; 996 as core genes, and 753 as variable. After analyzing the distribution of genomic variation, they determined that there was a greater amount of variation in the core resistance genes (Dolatabadian et al., 2020). A similar study in *Brassica* to develop a pangenome determined that 30 of the 53 accessions used to build the pangenome showed SV enrichment in regions of the genome associated with stress, defense, and auxin pathways (Samans et al., 2017). Fuentes et al. performed SV analysis on 3000 rice genomes and found SVs to be enriched in regions of the genome associated with stress response, including cell death, kinase activity, and nucleotide binding (Fuentes et al., 2019).

All these studies strengthen the association between structural variation and plant disease response, and represent the potential opportunity for utilizing SVs as a means to manipulate plant disease response. Additionally, there is a growing body of research associating specific SVs with disease resistance. A copy number variation in the *Rhg1* gene in soybean has been associated with nematode resistance (Cook et al., 2012; Bayless et al., 2016; Bayless et al., 2018; Bayless et al., 2019). Similarly, a copy number variation in the thaumatin-like protein (TLP) in Scott’s pine is associated with root rot resistance against the fungus *Heterobasidion annosum* (Skipars et al., 2012). A presence absence variation in *Brassica napus* is linked to *Verticillium longisporum* resistance (Gabur et al., 2020).

### Structural variation impacts tomato disease responses

Studies to investigate the impact of SVs on tomato disease resistance have been relatively limited compared to other crop plants such as maize, soybean, or rice. However, advances in sequencing technology and genomic assembly have allowed researchers to capture SVs across the entire genome, rather than SVs in a specific gene. Wang et al. created a high-quality genome for the wild relative of tomato, *S. pimpinellifolium*, and discovered over 92,000 SVs when compared to a modern tomato variety (Wang et al., 2020). They were further able to associate 14.8% of indels with coding or promoter regions, and SVs appeared to be enriched in regions of the genome associated to metabolic processes, signaling, reproduction, or stimuli response, and often associated with disease resistance. From this analysis, they concluded that SVs in *S. pimpinellifolum* may play a critical role in fruit quality and disease response.

Nucleotide variation in *R* genes at specific positions can have dramatic effects on the activity of disease resistance proteins. Nucleotide changes in the leucine-rich repeat receptor (LRR) *Flagellin sensing 3* (*Fls3*), which detects the bacterial flagellin peptide flgII-28, can lead to dramatic changes in Fls3 function (Hind et al., 2016; Roberts et al., 2020; Roberts et al., 2019). While the *Fls3* gene is present in most tomato accessions, genomic variants that lead to changes in the amino acid sequence can impair the ability for tomato to recognize the flgII-28 peptide across accessions. While the full effects of structural variation are yet to be revealed, it is clear that even small structural changes in Fls3 could greatly impact its function. Swapping inner-juxtamembrane domains between tomato flagellin receptors Fls2 and Fls3 caused a complete loss of in vitro kinase activity and reactive oxygen species (ROS) production in transient assays expressing chimeric Fls2/Fls3 constructs in leaves (Roberts et al., 2020). Within the kinase domain, the GxGxxG motif in subdomain I, which is involved in ATP binding and kinase activity, is conserved in Fls2 but not Fls3. Fls3 has stronger kinase activity in vitro that is associated with changes to the subdomain I motif (GxSxxS), and changes in this motif result in reduced kinase activity for Fls3 (Roberts et al., 2020). This is also apparent for the tomato *Flagellin sensing 2* (*Fls2*) paralog, *Fls2.2*, which is located approximately 3.8 kb away from *Fls2.1* and likely arose from a tandem gene duplication event (Roberts et al., 2019; Jacobs et al., 2017). A CRISPR/Cas9 knockout mutation in Fls2.1 causes a complete loss of flg22 recognition in tomato, suggesting that Fls2.1 is the only functional Fls2 in tomato (Jacobs et al., 2017).

*R* genes are the most divergent gene family in plants, and copy number variation in NBS-LRRs is likely an advantageous mechanism for plants to maintain diverse genes following duplication events to adapt to different and/or novel pathogens (Wei et al., 2016; Andolfo et al., 2021). Structural variation in *R* gene blocks leads to genomic variability and *R* gene diversification through chromosomal rearrangements, transposable elements, insertions, deletions, and nucleotide polymorphisms. Compared to other plant families, the *Solanaceae* have a large *R* gene copy number variation, with 87 *R* gene subfamilies described with significant variation in intron gains and losses (Andolfo et al., 2021). In a population of a wild relative of tomato, *Solanum pennellii,* a comparison of diverse and less diverse *S. pennellii* populations showed that even the less diverse populations still maintained genomic diversity and nucleotide polymorphisms in specific R genes (Stam et al., 2016). One example of LRR copy number variation and its impact on disease resistance is of the tomato *Cladosporium fulvum* resistance genes Cf*-2* and Cf*-5,* which differ in their LRR copy numbers due to recombination events. LRR copy numbers which range from 25 to 38 leucine-rich repeats and encode race-specific resistance against *C. fulvum (Dixon et al.,* 1998*)*. Additionally, the coiled-coil nucleotide-binding subclass of NLRs (CNLs) in tomato and five of its wild relatives were recently found to harbor structural variation in their N-termini in the form of extended CNLs (exCNL) that arose from tandem duplications of exon segments (Seong et al., 2020). Efforts to determine the effects of structural variation on R genes are underway, including a published tool to search for polymorphisms associated with NBS-LRR genes in potato (*Solanum tuberosum)* (Prakash et al., 2020). Table 2 summarizes known tomato structural variants and their associated phenotypes.

**Table 2:** Examples of known structural variants in tomato

SV gene	Type of SV	Associated Phenotype	References
Various	Various	Tomato domestication	(Blanca et al., 2015; Lin et al., 2014)
*sb1/sb3*	Tandem duplication	Changes in tomato branching patterns	(Soyk et al., 2019a; Soyk et al., 2019b)
*fas*	Large inversion	Increased fruit size	(Xu et al., 2015)
*CSR*	Deletion	Increased fruit weight	(Mu et al., 2017)
*LNK2*	Deletion	Change in circadian rhythm	(Muller et al., 2018)
*Fls3*	SNPs, domain swaps	Loss of function	(Hind et al., 2016; Roberts et al., 2020; Roberts et al., 2019)
*Fls2.2*	Tandem duplication of *Fls2.1*(paralog)	Loss of function	(Roberts et al., 2019; Jacobs et al., 2017)
Cf*-2* and Cf*-5*	Copy number variation	Race-specific resistance	(Dixon et al., 1998)
exCNLs	Tandem duplications	Subclass of NLRs in tomato and its wild relatives with extended N-termini	(Seong et al., 2020)

### Engineering immunity in tomato using SVs

Although the technology to sequence and analyze genomic structural variants has dramatically evolved through the decades, it remains difficult to recreate positive impacts of SVs using genetic engineering, such as the recognition and defense against pathogens (immunity). By definition, SVs are larger than other forms of genomic variation. This presents a problem when current CRISPR protocols for plants have been optimized for smaller regions of genetic material. Zhang et al. generated 245 T0 CRISPR/Cas9 events that induced mutations in 63 immunity-associated genes, and evaluated the efficiency of different CRISPR systems to induce mutations (Zhang et al., 2020). They found that 87% of mutations in tomato using the CRISPR-Cas9 system were 10 base pairs or less. However, it was possible to create an insertion greater than 50 bp, or a deletion larger than 400 bp. In mammalian systems, the Type 1-E CRISPR system has been shown to introduce deletions up to 100 kilobases. Currently, the largest mutations engineered in plants have been performed using the CRISPR/Cas9 system in tandem with multiple guide RNAs (Cai et al., 2018). Using this system, researchers were able to introduce deletions ranging from 599 to 1618 bp with 15.6% frequency, and fragments exceeding 4.5 kb with 12.1% frequency.

There is also the potential to use CRISPR/Cas Prime editing to genetically engineer changes in structural variations. Prime editing vectors for plants have been developed, but with overall low mutation efficiency with the highest being 53.2% for maize (Jiang et al., 2020). In tomato, Prime editing mutation efficiency is very low with the highest reported efficiency of 1.66% (Lu et al., 2021). However, a 66 bp insertion was successfully reported that enabled split-GFP fluorescent tagging in Arabidopsis protoplasts (Wang & Chen, 2020). While there are still efforts needed to make Prime editing more efficient in plants, including tomato, these preliminary studies demonstrate promise for gene editing structural variants. A resource database to provide information about plants generated with CRISPR mutations (Plant Genome Editing Database) (Zheng et al., 2019) has been developed as a means to view and track current CRISPR-generated plants and request seeds and/or materials from the authors. The availability and shared nature of these CRISPR lines makes resources available to scientists who wish to screen CRISPR plants for their phenotype of interest. Generating large structural variants in the future holds promise for multiple-use research projects if seeds are made available to publicly-funded research.

## Concluding remarks and future directions

There is still much to learn about how structural variation affects plants, but the evidence thus far reveals a strong association with structural variation and disease responses. Recent innovations in genomic tools and the development of large collections of genomic resources has led to great insights into the discovery of structural variants in plants, molecular mechanisms of genetics, and the effects of genomic changes on disease resistance or development. Additionally, improvements in gene editing tools such as CRISPR pave the way for ‘designer genomes’ to improve agricultural germplasm in the future. Such improvements could allow scientists to better and more quickly adapt to the adverse impacts of climate change, including emerging pathogens and biotic stresses, abiotic stresses, increased need for biofuel production, and a significant need for carbon sequestration from the atmosphere.

## Data Availability

Not applicable.

## Abbreviations

SV: Structural variant
BRCA1/BRCA2: Breast cancer 1/2
SNV: Single nucleotide variation
UTR: untranslated region
SNPs: Single nucleotide polymorphism
SRS: short read sequencing
Array CGH: array comparative genomic hybridization
LRS: long read sequencing
SMRT: Single molecule real time sequencing
CNV: copy number variation
VRN-A1: vernalization- A1
BOR1: Boron efflux transporter 1
MATE1: Multidrug and toxin extrusion protein 1
*sb1/sb*3: suppressor of branching 1/3
*fas*: natural mutation in *CLAVATA* pathway leading to increased fruit size
*CSR*: Cell Size Regulator
*LNK2*: Night light-inducible and clock-regulated gene 2
CRISPR: Clustered Regularly Interspaced Short Palindromic Repeats
*Rhg1*: Resistance to *Heterodera glycines* 1
TLP: thaumatin-like protein
LRR: leucine rich repeats
Fls3: Flagellin sensing 3
ROS: reactive oxygen species
Fls2: Flagellin sensing 2
NBS-LRR: nucleotide binding site-leucine rich repeats
